# Guillain–Barré syndrome mimics

**DOI:** 10.1002/brb3.960

**Published:** 2018-04-10

**Authors:** Sai Leong Tham, Kalpana Prasad, Thirugnanam Umapathi

**Affiliations:** ^1^ The School of Medicine, Medical Sciences and Nutrition University of Aberdeen Aberdeen UK; ^2^ Department of Neurology National Neuroscience Institute Singapore City Singapore

**Keywords:** : acute flaccid paralysis, acute polyradiculoneuropathy, Brighton Collaboration criteria for Guillain–Barré syndrome, Guillain–Barré syndrome, mimics of Guillain–Barré syndrome

## Abstract

The Brighton Collaboration criteria have standardized the clinical and laboratory‐supported diagnosis of Guillain–Barré syndrome (GBS) and Miller Fisher syndrome (MFS) in a way that is applicable in many parts of the world with variable resources. The caveat within the criteria, “absence of an identified alternative diagnosis for weakness” makes GBS a diagnosis of exclusion. Accurate diagnosis of GBS requires a good understanding of an updated, locally contextualised list of mimics, and features that distinguish them from GBS.

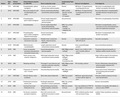

## 1 INTRODUCTION

The diagnosis of Guillain–Barré syndrome (GBS), the leading global cause of acute flaccid paralysis (Yuki & Hartung, 2012), is largely based on clinical features, and supported by serological, electrodiagnostic, and immunological investigations. However, the manifestations of GBS are protean and include limited forms such as acute ophthalmoparesis (Wakerley, Uncini, & Yuki, 2014) and pharyngeal–cervical–brachial variant (Wakerley & Yuki, 2013). Central nervous system signs such as drowsiness, hyper‐reflexia, and Babinski reflex can rarely be present, indicating the related Bickerstaff encephalitis (Odaka et al., 2003). Hence, a good appreciation of common GBS mimics is necessary for the accurate diagnosis of GBS; as articulated by the Brighton Collaboration criteria, “absence of an identified alternative diagnosis for weakness” (Sejvar et al., 2011). The list of disorders that is commonly mistaken for GBS varies with the setting. The aim of this study, performed at National Neuroscience Institute, Singapore, was to describe the clinical features and investigations of patients who were initially diagnosed as GBS but subsequent evaluation led to the diagnosis of an unrelated disorder that mimicked GBS.

In our institution, a tertiary neurological institute, GBS patients have been prospectively and continuously data‐based since early 2010. Of a total hundred and forty‐one patients, twelve turned out to have a different diagnosis during follow‐up (Table 1, Patients 1‐12). Three additional patients (Table 1, Patients 13‐15) were identified prior to the start of the database. A retrospective record review study was performed on these fifteen patients who presented from January 2008 to September 2014. This study was approved by our Institutional Review Board.

**Table 1:** Demographic data, clinical presentation, investigations and final diagnosis of GBS mimics

Patient	Age/Sex	Clinical presentation	Clinical features atypical for GBS/MFS	Nerve conduction study	Cerebrospinal fluid (CSF)	Relevant investigations	Final diagnosis
1	27/F	MFS/GBS	Encephalopathy, upbeat nystagmus	Acute‐on‐chronic axonal sensorimotor polyneuropathy	Normal	MRI Brain: T2 hyperintensity in periaqueductal area	Wernicke's encephalopathy with beriberi neuropathy, Alcoholism
2	29/M	MFS/GBS	Recurrent weakness with encephalopathy, pes cavus	Chronic axonal‐demyelinating sensorimotor polyneuropathy	WBC, protein, glucose: normal. Lactate: 3.7 mmol/L	MRI Brain: T2 hyperintensity in periaqueductal area and thalami	Recurrent Wernicke's encephalopathy (possible thiamine transporter gene defect)
3	49/M	MFS/GBS	Encephalopathy, gaze‐evoked nystagmus	Chronic length‐dependent axonal sensorimotor polyneuropathy	Not performed		Wernicke's encephalopathy, Alcoholism
4	65/F	MFS/GBS	Encephalopathy	Chronic length‐dependent axonal‐demyelinating sensorimotor polyneuropathy	WBC 2/μl, protein **0.67 g/L,** cytology: no malignant cell		Wernicke's encephalopathy; serous carcinoma of ovary and endometrium with intra‐abdominal metastasis
5	61/F	MFS/GBS	Encephalopathy, gaze‐evoked nystagmus, impaired vertical gaze	Axonal sensorimotor polyneuropathy	Normal		Wernicke's encephalopathy with beriberi neuropathy
6	54/M	GBS	Asymmetric weakness and sensory loss; pain	Multiple motor conduction blocks, reduced sensory potentials	Normal	Serum cryoglobulin: positive, ESR 134 mm/hr	Vasculitic neuropathy
7	25/M	MFS/GBS	Abdominal pain, encephalopathy	Length‐dependent axonal sensorimotor polyneuropathy	Normal	Urine: porphobilinogen present	Acute intermittent porphyria
8	35/M	GBS	Acute maculopathy, persistent dysautonomia and painful sensory loss	Axonal‐demyelinating polyneuropathy	WBC **90/μl**, protein **1.27 g/L**	HbA1C from 12.8% to 5.9% over 3 months while on insulin	Treatment‐induced neuropathy of diabetes mellitus (insulin neuritis)
9	57/M	GBS	Patchy sensory loss, systemic malignancy	Mild length‐dependent axonal sensorimotor polyneuropathy	WBC **79/μl**, protein **2.99 g/L**, cytology: B‐lymphoma cells		Stage 4 diffuse large B‐cell lymphoma with leptomeningeal metastasis
10	28/M	GBS	Relapsing remitting	Prolonged F‐wave latencies and reduced conduction velocities in motor nerves	WBC **10/μl**, protein **0.53 g/L**	HIV serology: positive	Chronic inflammatory demyelinating polyneuropathy related to HIV seroconversion
11	51/M	GBS	Progression of weakness over >4 weeks	Demyelinating polyneuropathy	WBC **9/μl**, protein **>3.00 g/L**		Acute‐onset chronic inflammatory demyelinating polyneuropathy (A‐CIDP)
12	53/F	MFS/GBS	Normal reflexes, static symptoms	Blink reflex absent bilaterally	WBC 0/μl, protein **0.60 g/L**	MRI Brain: signal abnormality in facial colliculi region of pons	Clinically isolated syndrome of brain stem demyelination
13	37/M	GBS	Predominant persistent sensory loss	Nonlength‐dependent reduction in sensory potentials	WBC 3/μl, protein **1.01 g/L**		Sensory neuronopathy
14	19/M	GBS	Asymmetrical onset, thickened nerves	Demyelinating sensorimotor polyneuropathy, conduction blocks	WBC 0/μl, protein 0.25 g/L	One copy (deletion) of PMP 22 gene region on chromosome 17	Hereditary neuropathy with liability to pressure palsy (HNPP)
15	41/M	GBS	No sensory symptoms, persistent weakness	Short (10 s) exercise test: significant increment in motor potentials	Not performed	VGCC antibody positive, Small cell carcinoma of lung	Lambert–Eaton myasthenic syndrome (LEMS)

GBS, Guillian–Barre syndrome; MFS, Miller Fisher syndrome; WBC, white blood cell; MRI, magnetic resonance imaging; ESR, erythrocyte sedimentation rate; HIV, human immunodeficiency virus; VGCC, voltage‐gated calcium channel; HNPP, hereditary neuropathy with liability to pressure palsy.

The abnormal values of cerebrospinal fluid are indicated in bold. Cerebrospinal fluid normal ranges: WBC: 0 – 5 cells/μl, protein: 0.10 – 0.40 g/L, glucose: 2.5 – 5.5 mmol/L.

For each patient in Table 1, we have listed the salient features that resembled GBS and Miller Fisher syndrome (MFS) and those that eventually led to the correct diagnosis. There were a total of eleven disorders that mimicked GBS.

### 1.1 Patient 1

A 27‐year‐old woman presented with rapidly progressive tetraparesis over 3 days. She had ophthalmoplegia, upbeating nystagmus, bifacial weakness, bulbar palsy, flaccid tetraparesis, areflexia, and distal sensory loss. She was diagnosed as GBS overlapping with MFS. Two days later, she was encephalopathic. Nerve conduction study (NCS) showed severe, diffuse, acute‐on‐chronic, axonal sensorimotor polyneuropathy. Magnetic resonance imaging (MRI) brain showed restricted diffusion in both caudate heads, and T2 hyperintensity in the periaqueductal area and bilateral dorsomedial thalamus. Cerebrospinal fluid (CSF) analysis was normal. A history of chronic alcoholism was elicited. She was diagnosed as Wernicke's encephalopathy with beriberi neuropathy. She recovered fully with intravenous thiamine treatment.

### 1.2 Patient 6

A 54‐year‐old man had pain, numbness, and weakness in limbs over 2 days. He had asymmetrical flaccid weakness in limbs with reduced reflexes and patchy sensory loss. He progressed to symmetrical weakness in all limbs. CSF was normal. NCS within a few days of symptoms showed multiple motor conduction blocks and reduced sensory potentials. He was diagnosed with GBS. He did not improve with intravenous immunoglobulin (IVIg). NCS at the third week showed inexcitable nerves. The asymmetric painful weakness raised the possibility of mononeuritis multiplex. Further investigations revealed a diagnosis of vasculitic neuropathy associated with cryoglobulinemia. His weakness gradually improved with immunosuppressant drugs.

### 1.3 Patient 7

A 25‐year‐old man presented with abdominal pain, tea‐colored urine, altered mental state, and seizure. He was treated with intravenous phenytoin. Two days later, he developed flaccid tetraparesis and ophthalmoplegia. MRI brain showed mildly enhancing T2 and diffusion‐weighted imaging (DWI) hyperintensities in bilateral caudate nucleus heads, periaqueductal gray matter, mammillary bodies, superior and inferior colliculi, bilateral hypoglossal and vestibular nuclei. CSF was normal. NCS showed length‐dependent axonal sensorimotor polyneuropathy. His urine ketones were raised as he was not fed on account of the abdominal pain. He was treated with intravenous thiamine for Wernicke's encephalopathy with improvement in ophthalmoplegia and MRI brain findings. However, he continued to have flaccid weakness, hyporeflexia, autonomic dysfunction, and required mechanical ventilation. GBS overlapping with MFS was considered and he was treated with IVIg. He did not improve. He was finally diagnosed with acute intermittent porphyria (AIP) when the urine test was positive for porphobilinogen. All medications that can worsen AIP were withdrawn. Patient eventually improved to premorbid status. We believe the MRI changes were due to concomitant Wernicke's encephalopathy.

### 1.4 Patient 8

A 35‐year‐old man was started on insulin therapy for newly diagnosed diabetes mellitus. HbA1c decreased from 12.8% to 5.9% over 3 months. He developed maculopathy, central retinal vein occlusion, orthostatic hypotension, supraventricular tachycardia, hypoglycemia spells, and recurrent syncope. Four days after an episode of Group B Salmonella gastroenteritis, he developed flaccid paraparesis, areflexia, retention of urine, and distal painful sensory loss. He was diagnosed as GBS. He was treated with IVIg. NCS showed axonal‐demyelinating polyneuropathy. CSF showed elevated white blood cell (WBC) count (90 cells/μl) and raised protein (1.27 g/L) with normal glucose. The diagnosis was revised to treatment‐induced neuropathy of diabetes mellitus because of persistent and progressive small fiber neuropathy and dysautonomia many weeks later. He gradually improved with optimization of blood sugar and neuropathic pain medications.

### 1.5 Patient 9

A 57‐year‐old man with stage four diffuse large B‐cell lymphoma, treated with eight cycles of rituximab, cyclophosphamide, doxorubicin, vincristine, prednisolone (RCHOP) therapy over 5 months, presented with acute‐onset right facial weakness, bulbar palsy, flaccid weakness in lower limbs with areflexia, and distal sensory loss in all limbs. He was diagnosed as GBS and treated with IVIg. NCS showed mild length‐dependent axonal sensorimotor polyneuropathy. CSF showed raised WBC count (79 cells/μl), elevated protein (2.99 g/L), and reduced glucose (2.1 mmol/L). CSF cytology showed B‐lymphoma cells. His diagnosis was revised to diffuse large B‐cell lymphoma with leptomeningeal metastasis. He was transferred to a cancer center for treatment of lymphoma.

### 1.6 Patient 10

A 28‐year‐old man presented with acute‐onset flaccid tetraparesis with hyporeflexia. NCS showed prolonged F‐wave latencies and nonuniform slowing of motor nerves consistent with GBS. CSF showed raised WBC count (10 cells/μl), mildly elevated protein (0.53 g/L), and normal glucose. HIV serology was positive. He recovered fully after IVIg treatment. In the next 1 year, he had few more episodes of weakness that resolved with IVIg and eventually resolved with highly active antiretroviral treatment. His final diagnosis was chronic inflammatory demyelinating polyneuropathy related to HIV seroconversion.

### 1.7 Patient 11

A 51‐year‐old man presented with difficulty walking for 2 weeks that was preceded by upper respiratory tract infection. He had symmetrical tetraparesis and reduction in reflexes. Cranial nerves were normal. CSF showed raised WBC count (9 cells/μl), raised protein (>3.00 g/L) and normal glucose. NCS showed demyelinating polyneuropathy. He was diagnosed with GBS, and IVIg was administered. A month later, he was readmitted with progressive tetraparesis, areflexia, and distal sensory loss. His diagnosis was revised to acute‐onset chronic inflammatory demyelinating polyneuropathy (A‐CIDP). His condition eventually improved with corticosteroid and mycophenolate mofetil.

### 1.8 Patient 12

A 53‐year‐old woman presented with vertiginous giddiness and unsteady gait for 2 weeks. She was treated with steroid prior to coming to our center. Clinical examination showed complex ophthalmoparesis, and bilateral lower motor neuron facial weakness. There was mild left upper limb dysmetria and she could not tandem walk. She had normal power, reflexes, and sensation. She was diagnosed with GBS overlapping with MFS. NCS showed only absent bilateral blink reflex. CSF showed elevated protein (0.60 g/L), no pleocytosis, and normal glucose. However, MRI brain showed nonenhancing signal abnormalities in the facial colliculi region of pons, periventricular white matter, and subcortical white matter. Final diagnosis was clinically isolated syndrome of brain stem demyelination. Three months later, she only had mild residual left facial weakness.

### 1.9 Patient 13

A 37‐year‐old man with systemic lupus erythematosus (SLE), presented with acute‐onset of severe loss of sensation associated with areflexia in all limbs and inability to walk. He was diagnosed with GBS and treated with IVIg. CSF showed normal WBC count (3 cells/μl) and raised protein (1.01 g/L). NCS within 1‐week from the onset was normal but subsequent study in the second week detected nonlength‐dependent reduction in sensory potentials. His mobility improved over few weeks but continued to have sensory loss and persistent postural hypotension. Anti‐Ro (SSA) was weakly positive. He was finally diagnosed as subacute sensory neuronopathy from underlying Sjogrens‐SLE. With escalation of immunosuppression, he gradually improved but he remains disabled by severe sensory ataxia and orthostatic hypotension.

### 1.10 Patient 14

A 19‐year‐old man undergoing military recruitment developed acute sequential weakness and numbness of his limbs during a field military camp, where he had to sleep and assume various prone postures on hard grounds for prolonged periods. He had symmetric flaccid weakness and patchy sensory loss. Cranial nerves were normal. NCS showed multiple conduction blocks, prolonged distal motor latencies and F‐wave latencies, and reduced sensory potentials. CSF analysis was normal. He was diagnosed with GBS and treated with IVIg. With the additional finding of thickened nerves, hereditary neuropathy with liability to pressure palsy (HNPP) was considered. Further enquiry revealed a possible family history of HNPP. One copy (deletion) of the PMP22 gene region on chromosome 17 was demonstrated. We cannot exclude the possibility of GBS occurring in a patient with HNPP; the absence of raised CSF protein in second week of illness points against it (Crum, Sorenson, Abad, & Dyck, 2000; Korn‐Lubetzki, Argov, Raas‐Rothschild, Wirguin, & Steiner, 2002).

### 1.11 Patient 15

A 41‐year‐old man, chronic smoker, presented with limb weakness for 3 weeks that remained fairly static. He had mild bilateral ptosis, proximal weakness, areflexia, and normal sensation. He was diagnosed with GBS in another hospital. On review, the reflexes were noted to improve with exercise. NCS showed reduced distal compound muscle action potential (CMAP) amplitudes that increased after short (10 s) exercise test and decreased during 3‐Hertz repetitive nerve stimulation test. He was diagnosed with Lambert–Eaton myasthenic syndrome (LEMS). Voltage‐gated calcium channel (VGCC) antibody was present. Computed tomography (CT) of the thorax showed left lung suprahilar mass which on biopsy revealed small cell carcinoma. He was treated with plasma exchange and 3,4‐diaminopyridine. He also received chemotherapy and radiotherapy for small cell lung carcinoma. His weakness improved after 6 months.

## 2 DISCUSSION

We have listed the conditions that were mistaken for GBS in a tertiary care hospital of a developed country. We would like to highlight the following general practical points that are useful in distinguishing them from GBS.

Unless Bickerstaff encephalitis is in consideration, altered mental state should not be present in GBS. The temporal evolution of neurological deficits and severe deficits that remain without significant recovery (Patients 6, 7, 8, and 15), or presentation of relapsing pattern (Patients 2, 10, and 11) should prompt a review of diagnosis. Sural sparing pattern of sensory abnormality, defined as a greater decrease in median and or ulnar sensory nerve action potential (SNAP) compared to that of the sural SNAP, was present in only one patient with GBS mimic (patient 13). He had sensory neuronopathy and sural sparing pattern was present in the NCS performed on Day 8. This corroborates the observation of Derksen et al. (2014) that sural sparing pattern can help discriminate GBS from its mimics.

Cytoalbuminologic dissociation in spinal fluid examined about 1 week after symptoms is useful in the diagnosis of GBS (Wong et al., 2015). However, the Brighton criteria use a CSF total white cell count of ˂50 cells/μl, considerably above the cutoffs used in many centers and originally proposed by Asbury and Cornblath (1990). Thirteen of our fifteen GBS mimics patients had CSF examination (performed at a median of 8 days). Five (Patients 4, 10, 11, 12, and 13) fulfilled criteria for cytoalbuminologic dissociation in concordance with the Brighton criteria. Using our laboratory normal value of less than 6 cells/μl, only three patients had cytoalbuminologic dissociation (Patients 4, 12, and 13). Spinal fluid examination performed at an appropriate time, and using stricter criteria than that proposed in Brighton criteria can help the accuracy of GBS diagnosis.

The spectrum of disorders that resembles GBS evolves with changes in the practice of medicine, patient demographics, and disease epidemiology. Local setting should also be considered. For instance, we expect acute beriberi neuropathy to be less frequent in countries where alcohol consumption is low for cultural–religious reasons. In our center, improvements in access to laboratory investigations and neuroimaging have removed previously common differential diagnoses of GBS such as acute spinal shock and hypokalemic periodic paralysis.

In conclusion, accurate diagnosis of GBS requires a good understanding of an updated, locally contextualised list of mimics, and features that distinguish them from GBS.
